# Genomics and data science: an application within an umbrella

**DOI:** 10.1186/s13059-019-1724-1

**Published:** 2019-05-29

**Authors:** Fábio C. P. Navarro, Hussein Mohsen, Chengfei Yan, Shantao Li, Mengting Gu, William Meyerson, Mark Gerstein

**Affiliations:** 1 Program in Computational Biology and Bioinformatics, Yale University, Bass 432, 266 Whitney Avenue, New Haven, CT 06520 USA; 2 Department of Molecular Biophysics and Biochemistry, Yale University, Bass 432, 266 Whitney Avenue, New Haven, CT 06520 USA; 3 Department of Computer Science, Yale University, Bass 432, 266 Whitney Avenue, New Haven, CT 06520 USA; 4 Department of Statistics and Data Science, Yale University, Bass 432, 266 Whitney Avenue, New Haven, CT 06520 USA; 5 Department of Computer Science, Stanford University, Stanford, CA 94305 USA; 6 Department of Biomedical Data Sciences, Stanford University, Stanford, CA 94305 USA

## Abstract

Data science allows the extraction of practical insights from large-scale data. Here, we contextualize it as an umbrella term, encompassing several disparate subdomains. We focus on how genomics fits as a specific application subdomain, in terms of well-known 3 V data and 4 M process frameworks (volume-velocity-variety and measurement-mining-modeling-manipulation, respectively). We further analyze the technical and cultural “exports” and “imports” between genomics and other data-science subdomains (e.g., astronomy). Finally, we discuss how data value, privacy, and ownership are pressing issues for data science applications, in general, and are especially relevant to genomics, due to the persistent nature of DNA.

## Introduction

Data science as a formal discipline is currently popular because of its tremendous commercial utility. Large companies have used several well-established computational and statistical techniques to mine high volumes of commercial and social data [1]. The broad interest across many applications stirred the birth of data science as a field that acts as an umbrella, uniting a number of disparate disciplines using a common set of computational approaches and techniques [2]. In some cases, these techniques were created, developed, or established in other data-driven fields (e.g., astronomy and earth science). In fact, some of these disciplines significantly predate the formal foundation of data science and have contributed to several techniques to cope with knowledge extraction from large amounts of data.

Many scholars have probed the origins of data science. For example, in 1960 Tukey described a new discipline called data analysis, which some consider being a forerunner of data science. He defined data analysis as the interplay between statistics, computer science, and mathematics [3]. Jim Gray also introduced the concept of data-intensive science in his book *The Fourth Paradigm* [4], and discussed how the developments in computer science would shape and transform segments of science to a data-driven exercise. More practically, the maturation of modern data science from an amorphous discipline can be tracked to the expansion of the technology industry and its adoption of several concepts at the confluence of statistics and algorithmic computer science, such as machine learning [5]. Somewhat less explored is the fact that several applied disciplines have contributed to a collection of techniques and cultural practices that today comprise data science.

### Contextualizing natural science within the data science umbrella

Long before the development of formal data science, and even computer science or statistics, traditional fields of natural sciences established an extensive culture around data management and analytics. For instance, physics has a long history of contributions of several concepts that are now at the foundation of data science. In particular, physicists such as Laplace, Gauss, Poisson, and Dirichlet have led the way for the development of hypothesis testing, least squares fits, and Gaussian, Poisson, and Dirichlet distributions [6].

More recently, physics also has contributed new data techniques and data infrastructure. For example, Ulam originally invented the Monte Carlo sampling method while he was working on the hydrogen bomb [7] and Berners-Lee, from the CERN (European Organization for Nuclear Research), developed the World Wide Web [8] to enable distributed collaboration in particle physics. While most disciplines are now experiencing issues with rapid data growth [9, 10], we find it interesting that physics had issues with data management long before most disciplines. As early as the 1970s, for example, Jashcek introduced the term “information explosion” to describe the rapid data growth in astrophysics [11].

Fundamental contributions to data management and analytics have not been exclusive to physics. The biological sciences, perhaps most prominently genetics, also have significantly influenced data science. For instance, many of the founders of modern statistics, including Galton, Pearson, and Fisher, pioneered principal component analysis, linear regression, and linear discriminant analysis while they were also preoccupied with analyzing large amounts of biological data [6]. More recently, methods such as logistic regression [12], clustering [13], decision trees [14], and neural networks [15] were either conceptualized or developed by researchers focused on biological questions. Even Shannon, a central figure in information theory, completed a short PhD in population genetics [16].

### Genomics and data science

**Fig. 1:**
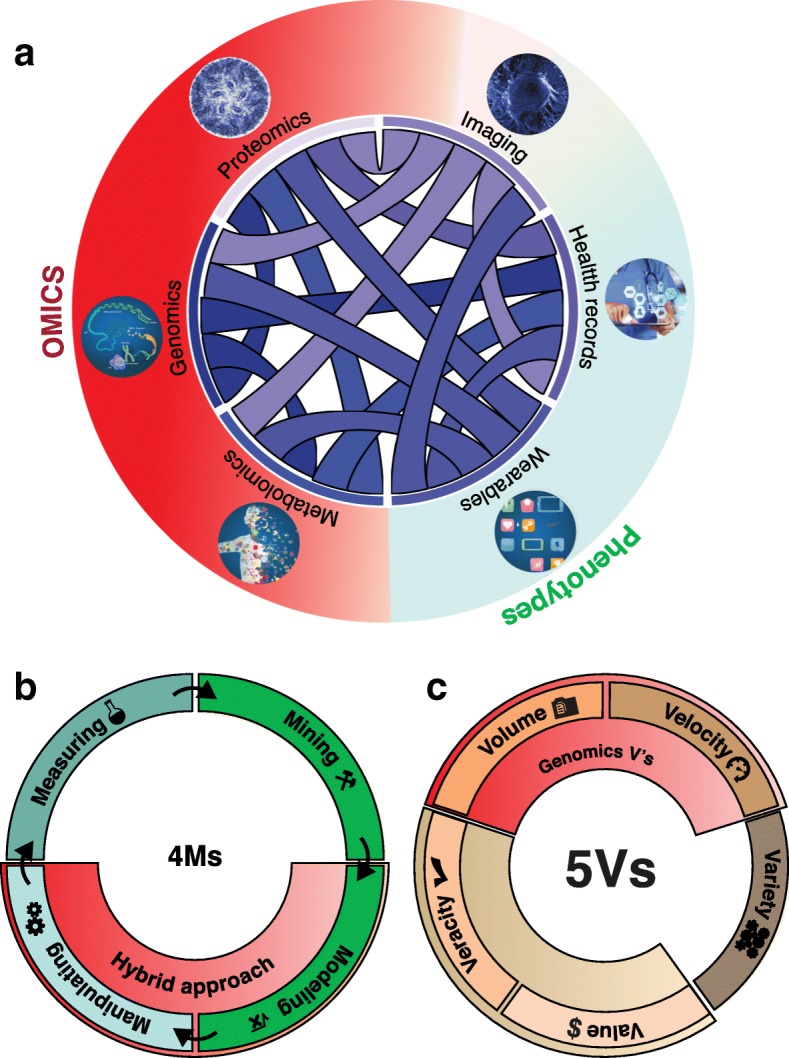
A holistic view of biomedical data science. **a** Biomedical data science emerged at the confluence of large-scale datasets connecting genomics, metabolomics, wearable devices, proteomics, health records, and imaging to statistics and computer science. **b** The 4 M processes framework. **c** The 5 V data framework

More recent biological disciplines such as macromolecular structure and genomics have inherited many of these data analytics features from genetics and other natural sciences. Genomics, for example, emerged in the 1980s at the confluence of genetics, statistics, and large-scale datasets [17]. The tremendous advancements in nucleic acid sequencing allowed the discipline to swiftly assume one of the most prominent positions in terms of raw data scale across all the sciences [18]. This pre-eminent role of genomics also inspired the emergence of many “-omics” terms inside and outside academia [19, 20]. Although today genomics is pre-eminent in terms of data scale, this may change over time due to technological developments in other areas, such as cryo-electron microscopy [21] and personal wearable devices [22]. Moreover, it is important to realize that many other existing data-rich areas in the biological sciences are also rapidly expanding, including image processing (including neuroimaging), macromolecular structure, health records analysis, proteomics, and the inter-relation of these large data sets, in turn, is giving rise to a new subfield termed biomedical data science (Fig. 1a).

Here, we explore how genomics has been, and probably will continue to be, a pre-eminent data science subdiscipline in terms of data growth and availability. We first explore how genomics data can be framed in terms of the 3Vs (data volume, velocity, and variety) to contextualize the discipline in the “big-data world”. We also explore how genomics processes can be framed in terms of the 4Ms (measurement, mining, modeling, and manipulating) to discuss how physical and biological modeling can be leveraged to generate better predictive models. Genomics researchers have been exchanging ideas with those from other data science subfields; we review some of these “imports” and “exports” in a third section. Finally, we explore issues related to data availability in relation to data ownership and privacy. Altogether, this perspective discusses the past, present, and future of genomics as a subfield of data science.

## Genomics versus other data science applications in terms of the V framework

One way of categorizing the data in data science disciplines is in terms of its volume, velocity, and variety. Within data science, this is broadly referred to as the V framework [23]. Over the years, the V framework has been expanded from its original 3Vs [24] (volume, velocity, and variety) to the most recent versions with four and five Vs (3 V + value and veracity; Fig. 1c) [25]. In general, the distinct V frameworks use certain data-related parameters to recognize issues and bottlenecks that might require a new set of tools and techniques to cope with unstructured and high-volume data. Here, we explore how we can use the original 3 V framework to evaluate the current state of data in genomics in relation to other applications in data sciences.

### Volume

**Fig. 2:**
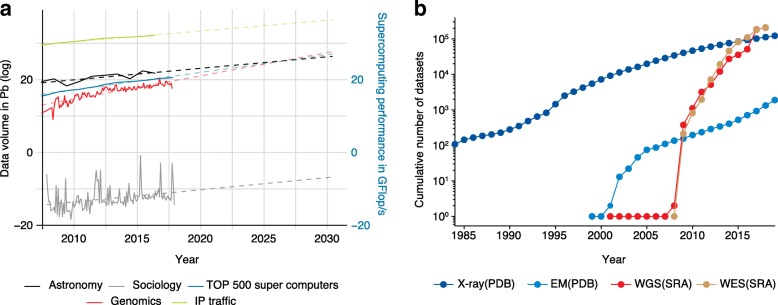
Data volume growth in genomics versus other disciplines. **a** Data volume growth in genomics in the context of other domains and data infrastructure (computing power and network throughput). *Continuous lines* indicate the amount of data archived in public repositories in genomics (SRA), astronomy (Earth Data, NASA), and sociology (Harvard dataverse). Data infrastructure such as computing power (*TOP500 SuperComputers*) and network throughput (*IPTraffic*) are also included. *Dashed lines* indicate projections of future growth in data volume and infrastructure capacity for the next decade. **b** Cumulative number of datasets being generated for whole genome sequencing (*WGS*) and whole exome sequencing (*WES*) in comparison with molecular structure datasets such as X-ray and electron microscopy (*EM*). *PDB* Protein Data Base, *SRA* Sequence Read Archive

One of the key aspects of genomics as a data science is the sheer amount of data being generated by sequencers. As shown in Fig. 2, we tried to put this data volume into context by comparing genomics datasets with other data-intensive disciplines. Figure 2a shows that the total volume of data in genomics is considerably smaller than the data generated by earth science [26], but orders of magnitude larger than the social sciences. The data growth trend in genomics, however, is greater than in other disciplines. In fact, some researchers have suggested that if the genomics data generation growth trend remains constant, genomics will soon generate more data than applications such as social media, earth sciences, and astronomy [27].

Many strategies have been used to address the increase in data volume in genomics. For example, researchers are now tending to discard primary data (e.g., FASTQ) and prioritizing the storage of secondary data such as compressed mapped reads (BAMs), variant calls (VCFs), or even only quantifications such as gene expression [28].

In Fig. 2b, we compare genomics to other data-driven disciplines in the biological sciences. This analysis clearly shows that the large amount of early biological data was not in genomics, but rather in macromolecular structure. Only in 2001, for example, did the number of datasets in genomics finally surpass protein-structure data. More recently, new trends have emerged with the rapidly increasing amount of electron microscopy data, due to the advent of cryo-electron microscopy, and of mass-spectrometry-based proteomics data. Perhaps these trends will shift the balance of biomedical data science in the future.

### Velocity

There are two widely accepted interpretations of data velocity: (i) the speed of data generation (Fig. 2) and (ii) the speed at which data are processed and made available [29].

We explored the growth of data generation in the previous section in relation to genomics. The sequencing of a human genome could soon take less than 24 h, down from 2 to 8 weeks by currently popular technologies and 13 years of uninterrupted sequencing work by the Human Genome Project (HGP) [30]. Other technologies, such as diagnostic imaging and microarrays, have also experienced remarkable drops in cost and complexity and, therefore, resulting data are much quicker to generate.

The second definition of data velocity speaks to the speed at which data are processed. A remarkable example is the speed of fraud detection during a credit card transaction or some types of high-frequency trading in finance [31]. In contrast, genomics data and data processing have been traditionally static, relying on fixed snapshots of genomes or transcriptomes. However, new fields leveraging rapid sequencing technologies, such as rapid diagnosis, epidemiology, and microbiome research, are beginning to use nucleic acid sequences for fast, dynamic tracking of diseases [32] and pathogens [33]. For these and other near-future technologies, we envision that fast, real-time processing might be necessary.

The description of the volume and velocity of genomics data has great implications for what types of computations are possible. For instance, when looking at the increase of genomics and other types of data relative to network traffic and bandwidth, one must decide whether to store, compute, or transfer datasets. This decision-making process can also be informed by the 3 V framework. In Fig. 2, we show that the computing power deployed for research and development (using the top 500 supercomputers as a proxy) is growing at a slower pace than genomic data growth. Additionally, while the global web traffic throughput has no foreseeable bottlenecks (Fig. 2a) [34], for researchers the costs of transferring such large-scale datasets might hinder data sharing and processing of large-scale genomics projects. Cloud computing is one way of addressing this bottleneck. Large consortia already tend to process and store most of their datasets on the cloud [35–37]. We believe genomics should consider the viability of public repositories that leverage cloud computing more broadly. At the current rate, the field will soon reach a critical point at which cloud solutions might be indispensable for large-scale analysis.

### Variety

**Fig. 3:**
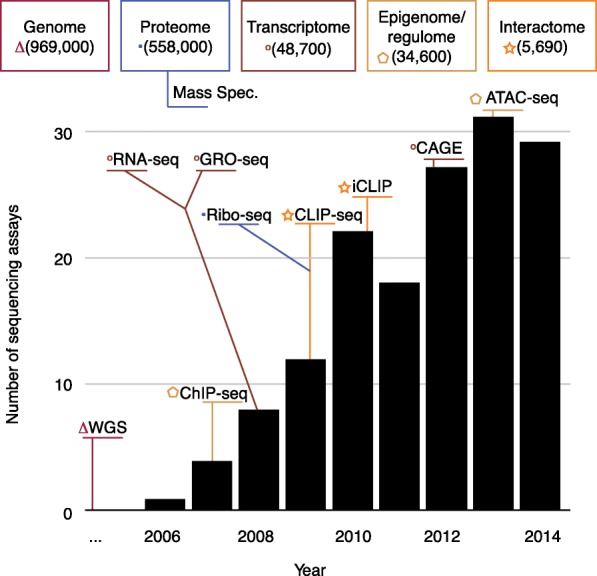
Variety of sequencing assays. Number of new sequencing protocols published per year. Popular protocols are highlighted in their year of publication and their connection to omes

Genomics data have a two-sided aspect. On one side is the monolithic sequencing data, ordered lists of nucleotides. In human genomics, traditionally these are mapped to the genome and are used to generate coverage or variation data. The monolithic nature of sequencing output, however, hides a much more varied set of assays that are used to measure many aspects of genomes. In Fig. 3 we illustrate this issue by showing the growth in the diversity of sequencing assays over time and displaying a few examples. We also display how different sequencing methods are connected to different omes [19]. The other side of genomics data is the complex phenotypic data with which the nucleotides are being correlated. Phenotypic data can consist of such diverse entities as simple and unstructured text descriptions from electronic health records, quantitative measurements from laboratories, sensors, and electronic trackers, and imaging data. The varied nature of the phenotypic data is more complicated; as the scale and diversity of sequencing data grow larger, more attention is being paid to the importance of standardizing and scaling the phenotypic data in a complementary fashion. For example, mobile devices can be used to harness large-scale consistent digital phenotypes [38].

## Genomics and the 4 M framework

Two aspects distinguish data science in the natural sciences from social science context. First, in the natural sciences much of the data are quantitative and structured; they often derive from sensor readings from experimental systems and observations under well-controlled conditions. In contrast, data in the social sciences are more frequently unstructured and derived from more subjective observations (e.g., interviews and surveys). Second, the natural sciences also have underlying chemical, physical, and biological models that are often highly mathematized and predictive.

Consequently, data science mining in the natural sciences is intimately associated with mathematical modeling. One succinct way of understanding this relationship is the 4 M framework, developed by Lauffenburger [39]. This concept describes the overall process in systems biology, closely related to genomics, in terms of (i) Measuring the quantity, (ii) large-scale Mining, which is what we often think of as data science, (3) Modeling the mined observations, and finally (4) Manipulating or testing this model to ensure it is accurate.

The hybrid approach of combining data mining and biophysical modeling is a reasonable way forward for genomics (Fig. 1b). Integrating physical–chemical mechanisms into machine learning provides valuable interpretability, boosts the data-efficiency in learning (e.g., through training-set augmentation and informative priors) and allows data extrapolation when observations are expensive or impossible [40]. On the other hand, data mining is able to accurately estimate model parameters, replace some complex parts of the models where theories are weak, and emulate some physical models for computational efficiency [41].

Short-term weather forecasting as an exemplar of this hybrid approach is perhaps what genomics is striving for. For this discipline, predictions are based on sensor data from around the globe and then fused with physical models. Weather forecasting was, in fact, one of the first applications of large-scale computing in the 1950s [42, 43]. However, it was an abject flop, trying to predict the weather solely based on physical models. Predictions were quickly found to only be correct for a short time, mostly because of the importance of the initial conditions. That imperfect attempt contributed to the development of the fields of nonlinear dynamics and chaos, and to the coining of the term “butterfly effect” [43]. However, subsequent years dramatically transformed weather prediction into a great success story, thanks to integrating physically based models with large datasets measured by satellites, weather balloons, and other sensors [43]. Moreover, the public’s appreciation for the probabilistic aspects of a weather forecast (i.e., people readily dress appropriately based on a chance of rain) foreshadows how it might respond to probabilistic “health forecasts” based on genomics.

## Imports and exports

Thus far, we have analyzed how genomics sits with other data-rich subfields in terms of data (volume, velocity, and variety) and processes. We argue that another aspect of genomics as an applied data science subfield is the frequent exchange of techniques and cultural practices. Over the years, genomics has imported and exported several concepts, practices, and techniques from other applied data science fields. While listing all of the movements is impossible in this piece, we will highlight a few key examples.

### Technical imports

A central aspect of genomics—the process of mapping reads to the human reference genome—relies on a foundational technique within data science: fast and memory-efficient string-processing algorithms. Protein pairwise alignment predates DNA sequence alignment. One of the first successful implementations of sequence alignment was based on Smith–Waterman [44] and dynamic programming [45, 46]. These methods were highly reliant on computing power and required substantial memory. With advances in other string-alignment techniques and the explosion of sequencing throughput, the field of genomics saw a surge in the performance of sequence alignment. As most sequencing technologies produce short reads, researchers generated several new methods using index techniques, starting around 2010. Several methods are now based on the Burrows–Wheeler transformation (BWA, bowtie) [47, 48], De Bruijn graphs (Kallisto, Salmon) [49, 50], and the Maximal Mappable Prefix (STAR) [51].

**Fig. 4:**
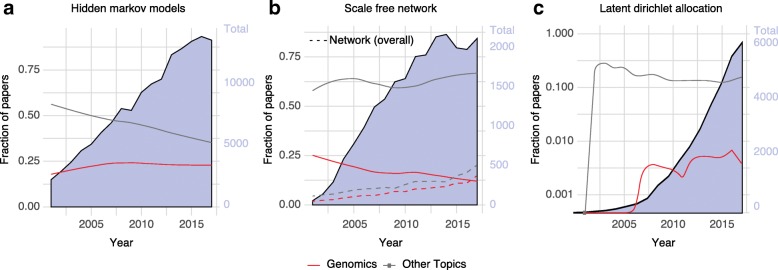
Technical exchanges between genomics and other data science subdisciplines. The background area displays the total number of publications per year for the terms. **a** Hidden Markov model, **b** Scale-free network, **c** latent Dirichlet allocation. *Continuous lines* indicate the fraction of papers related to topics in genomics and in other disciplines

Hidden Markov models (HMMs) are well-known algorithms used for modeling the sequential or time-series correlations between symbols or events. HMMs have been widely adopted in fields such as speech recognition and digital communication [52]. Data scientists also have long used HMMs to smooth a series of events in a varied number of datasets, such as the stock market, text suggestions, and in silico diagnosis [53]. The field of genomics has applied HMMs to predict chromatin states, annotate genomes, and study ancestry/population genetics [54]. Figure 4a displays the adoption of HMMs in genomics compared with other disciplines. It shows that the fraction of HMM papers related to genomics has been growing over time and today it corresponds to more than a quarter of the scientific publications related to the topic.

Another major import into genomics has been network science and, more broadly, graphs. Other subfields have been using networks for many tasks, including algorithm development [55], social network research [56], and modeling transportation systems [57]. Many subfields of genomics rely heavily on networks to model different aspects of the genome and subsequently generate new insights [58]. One of the first applications of networks within genomics and proteomics was protein–protein interaction networks [59]. These networks are used to describe the interaction between several protein(s) and protein domains within a genome to ultimately infer functional pathways [60]. After the development of large-scale transcriptome quantification and chromatin immunoprecipitation sequencing (ChIP-Seq), researchers built regulatory networks to describe co-regulated genes and learn more about pathways and hub genes [61]. Figure 4b shows the usage of “scale-free networks” and “networks” as a whole. While the overall use of networks has continued to grow in popularity in genomics after their introduction, the specific usage of scale-free has been falling, reflecting the brief moment of popularity of this concept.

Given the abundance of protein structures and DNA sequences, there has been an influx of deep-learning solutions imported from machine learning [62]. Many neural network architectures can be transferred to biological research. For example, the convolutional neural network (CNN) is widely applied in computer vision to detect objects in a positional invariant fashion. Similarly, convolution kernels in CNN are able to scan biological sequences and detect motifs, resembling position weight matrices (PWMs). Researchers are developing intriguing implementations of deep-learning networks to integrate large datasets, for instance, to detect gene homology [63], annotate and predict regulatory regions in the genome [64], predict polymer folding [65], predict protein binding [66], and predict the probability of a patient developing certain diseases from genetic variants [67]. While neural networks offer a highly flexible and powerful tool for data mining and machine learning, they are usually “black box” models and often very difficult to interpret.

### Cultural imports

The exchanges between genomics and other disciplines are not limited to methods and techniques, but also include cultural practices. As a discipline, protein-structure prediction pioneered concepts such as the Critical Assessment of protein Structure Prediction (CASP) competition format. CASP is a community-wide effort to evaluate predictions. Every 2 years since 1994, a committee of researchers has selected a group of proteins for which hundreds of research groups around the world will (i) experimentally describe and (ii) predict in silico its structure. CASP aims to determine the state of the art in modeling protein structure from amino acid sequences [68]. After research groups submit their predictions, independent assessors compare the models with the experiments and rank methods. In the most recent instantiation of CASP, over 100 groups submitted over 50,000 models for 82 targets. The success of the CASP competition has inspired more competitions in the biological community, including genomics. DREAM Challenges, for example, have played a leading role in organizing and catalyzing data-driven competitions to evaluate the performance of predictive models in genomics. Challenge themes have included “Genome-Scale Network Inference”, “Gene Expression Prediction”, “Alternative Splicing”, and “in vivo Transcription Factor Binding Site Prediction” [69]. DREAM Challenges was initiated in 2006, shortly before the well-known Netflix Challenge and the Kaggle platform, which were instrumental in advancing machine-learning research [70].

### Technical exports

A few methods exported from genomics to other fields were initially developed to address specific biological problems. However, these methods were later generalized for a broader set of applications. A notable example of such an export is the latent Dirichlet allocation (LDA) model. Pritchard et al. [71] initially proposed this unsupervised generative model to find a group of latent processes that, in combination, can be used to infer and predict individuals’ population ancestry based on single nucleotide variants. Blei, Ng, and Jordan [72] independently proposed the same model to learn the latent topics in natural language processing (NLP). Today, LDA and its countless variants have been widely adapted in, for example, text mining and political science. In fact, when we compare genomics with other topics such as text mining we observe that genomics currently accounts for a very small percentage of work related to LDA (Fig. 4c).

Genomics has also contributed to new methods of data visualization. One of the best examples is the Circos plot [73], which is related to the import above of network science. Circos was initially conceptualized as a circular representation of linear genomes. In its conception, this method displayed chromosomal translocations or large syntenic regions. As this visualization tool evolved to display more generic networks**,** it was also used to display highly connected datasets. In particular, the media has used Circos to display and track customer behavior, political citations, and migration patterns [73]. In genomics, networks and graphs are also being used in order to represent the human genome. For instance, researchers are attempting to represent the reference genome and its variants as a graph [74].

Another prominent idea exported from genomics is the notion of family classification based on large-scale datasets. This derives from the biological taxonomies dating back to Linnaeus, but also impacts the generation of protein and gene family databases [75, 76]. Other disciplines, for example, linguistics and neuroimaging, have also addressed similar issues by constructing semantic and brain region taxonomies [77, 78]. This concept has even made its way into pop culture; for example, Pandora initially described itself as the music genome project [79]. Another example is the art genome project [80], which maps characteristics (referred to as “genes”) that connect artists, artworks, architecture, and design objects across history.

### Cultural exports

Genomics has also tested and exported several cultural practices that can serve as a model for other data-rich disciplines [81]. On a fundamental level, these practices promote data openness and re-use, which are central issues to data science disciplines.

Most genomics datasets, and most prominently datasets derived from sequencing, are frequently openly accessible to the public. This practice is evidenced by the fact that most genomics journals require a public accession identifier for any dataset associated with a publication. This broad adoption of data openness is perhaps a reflection of how genomics evolved as a discipline. Genomics mainly emerged after the conclusion of HGP—a public initiative that, at its core, was dedicated to release a draft of the human genome that was not owned or patented by a company. It is also notable that the public effort was in direct competition with a private effort by Celera Genomics, which aimed to privatize and patent sections of the genome. Thus, during the development of the HGP, researchers elaborated the Bermuda principles, a set of rules that called for public releases of all data produced by HGP within 24 h of generation [82]. The adoption of the Bermuda principles had two main benefits for genomics. First, it facilitated the exchange of data between many of the dispersed researchers involved in the HGP. Second, perhaps due to the central role of the HGP, it spurred the adoption of open-data frameworks more broadly. In fact, today most large projects in genomics adopt Bermuda-like standards. For example, the 1000 Genomes [83] and the ENCODE [35] projects release their datasets openly before publication to allow other researchers to use their datasets [84]. Other subfields such as neuroscience (e.g., the human connectome) were also inspired by the openness and setup of the genomics community [81].

In order to attain a broad distribution of open datasets, genomics has also adopted the usage of central, large-scale public dataset repositories. Unlike several other applied fields, genomics data are frequently hosted on free and public platforms. The early adoption of these central dataset resources, such as the Sequence Read Archive (SRA), European Nucleotide Archive (ENA), GenBank, and Protein Data Base (PDB), to host large amounts of all sorts of genetics data, including microarray and sequencing data, has allowed researchers to easily query and promote re-use datasets produced by others [85].

The second effect of these large-scale central dataset repositories, such as the National Center for Biotechnology Information (NCBI) and ENA, is the incentive for early adoption of a small set of standard data formats. This uniformity of file formats encouraged standardized and facilitated access to genomics datasets. Most computations in genomics data are hosted as FASTA/FASTQ, BED, BAM, VCF, or bigwig files, which respectively represent sequences, coordinates, alignments, variants, and coverage of DNA or amino acid sequences. Furthermore, as previously discussed, the monolithic nature of genomic sequences also contributes to the standardization of pipelines and allows researchers to quickly test, adapt, and switch to other methods using the same input format [86].

**Fig. 5:**
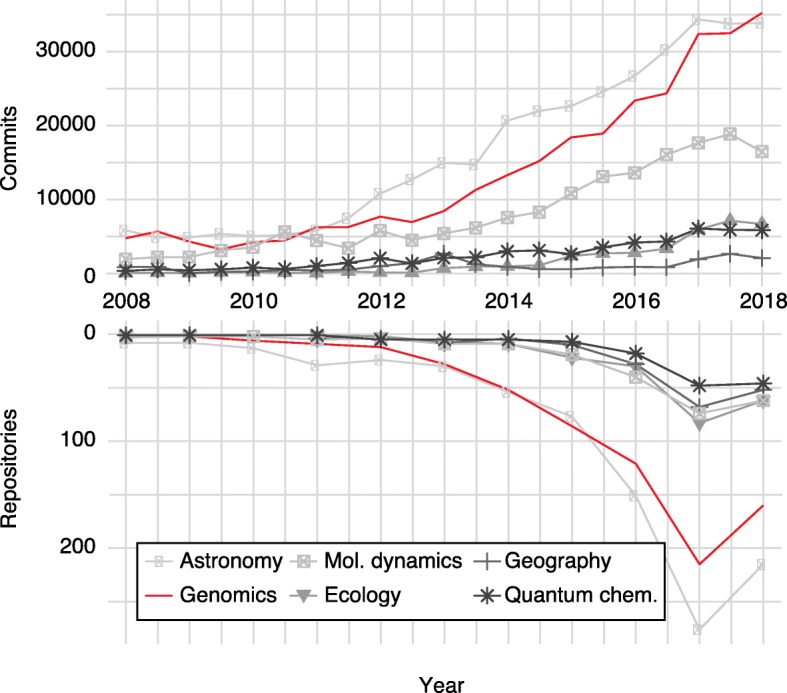
Open source adoption in genomics and other data science subdisciplines. The number of GitHub commits (*upper panel*) and new GitHub repositories (*lower panel*) per year for a variety of subfields. Subfield repositories were selected by GitHub topics such as genomics, astronomy, geography, molecular dynamics (*Mol. Dynamics*), quantum chemistry (*Quantum Chem.*), and ecology

The open-data nature of many large-scale genomics projects may also have spurred the adoption of open-source software within genomics. For example, most genomics journals require public links to source codes to publish in silico results or computational methods. To evaluate the adoption of open source in genomics, we used the growth of GitHub repositories and activity (commits) over time (Fig. 5). Compared with many fields of similar scale (e.g., astronomy and ecology) genomics has a particularly large representation on GitHub and this is growing rapidly.

## Data science issues with which genomics is grappling

### Privacy

In closing, we consider the issues that genomics and, more broadly, data science face both now and in the future. One of the major issues related to data science is privacy. Indeed, the current privacy concerns related to email, financial transactions, and surveillance cameras are critically important to the public [87]. The potential to cross-reference large datasets (e.g., via quasi-identifiers) can make privacy leaks non-intuitive [70]. Although genomics-related privacy overlaps with data science-related privacy, the former has some unique aspects given that the genome is passed down through generations and is fundamentally important to the public [88]. Leaking genomic information might be considered more damaging than leaking other types of information. Although we may not know everything about the genome today, we will know much more in 50 years. At that time, a person would not be able to take their or their children’s variants back after they have been released or leaked [88]. Finally, genomic data are considerably larger in scale than many other bits of individual information; that is, the genome carries much more individual data than a credit card or social security number. Taken together, these issues make genomic privacy particularly problematic.

However, in order to carry out several types of genomic calculations, particularly for phenotypic associations like genome-wide association studies, researchers can get better power and a stronger signal by using larger numbers of data points (i.e., genomes). Therefore, sharing and aggregating large amounts of information can result in net benefits to the group even if the individual’s privacy is slightly compromised. The Global Alliance for Genomics and Health (GA4GH) has made strides in developing technical ways to balance the concerns of individual privacy and social benefits of data sharing [89]. This group has discussed the notion of standardized consents associated with different datasets. The fields of security and privacy are undertaking projects like homomorphic encryption, where one can make certain calculations on an encrypted dataset without accessing its underlying contents [90].

### Data ownership

Privacy is an aspect of a larger issue of data ownership and control. Although the individual or patient typically is thought to own their personal data, a countervailing trend in biomedical research is the idea that the researcher who generates a dataset owns it. There is a longstanding tradition among researchers who have generated large datasets to progressively analyze their data over the course of several papers, even a career, to extract interesting stories and discoveries [91]. There is also the notion that human data, particularly health data, have obvious medical and commercial value, and thus companies and nations often seek ownership and control over large datasets.

From the data miner’s perspective, all information should be free and open, since such a practice would lead to the easy aggregation of a large amount of information, the best statistical power, and optimally mined results. Intuitively, aggregating larger datasets will, most frequently, give progressively better genotypes being associated to phenotypes.

Furthermore, even in an ideal scenario in which individuals consent to free access and the resulting dataset is completely open and freely shared by users, we imagine complications will arise from collection and sharing biases such as particular cohort ethnicity, diseases, and phenotypes being more open to share their genetic data. Socioeconomic status, education, and access to healthcare can all possibly cause skew in datasets, which would further bias mining efforts such as machine learning algorithms and knowledge extraction. For example, ImageNet, a heavily used dataset in image classification, has nearly half of the images coming from the USA. Similarly, about 80% of genome-wide association study catalog participants are of European descent, a group which only makes up 16% of the world population [92].

For this reason, completely open data sharing will probably not be reasonable for the best future genomic association studies. One possible technical solution for sharing genomics data might be the creation of a massive private enclave. This is very different from the World Wide Web, which is fundamentally a public entity. A massive private enclave would be licensed only to certified biomedical researchers to enable data sharing and provide a way to centralize the storage and computation of large datasets for maximum efficiency. We believe this is the most practical viewpoint going forward.

On the other hand, the positive externality of data sharing behaviors will become more significant as genomic science develops and becomes more powerful in aggregating and analyzing data. We believe that, in the future, introducing data property rights, Pigouvian subsidies, and regulation may be necessary to encourage a fair and efficient data trading and use environment. Furthermore, we imagine a future where people will grapple with complex data science issues such as sharing limited forms of data within certain contexts and pricing of data accordingly.

Lastly, data ownership is also associated with extracting profit and credit from the data. Companies and the public are realizing that the value of data does not only come from generating it per se, but also from analyzing the data in meaningful and innovative new ways. We need to recognize the appropriate approaches to not only recognize the generation of the data but also to value the analysis of large amounts of data and appropriately reward analysts as well as data generators.

## Conclusion

In this piece, we have described how genomics fits into the emergence of modern data science. We have characterized data science as an umbrella term that is increasingly connecting disparate application subdisciplines. We argue that several applied subdisciplines considerably predate formal data science and, in fact, were doing large-scale data analysis before it was “cool”. We explore how genomics is perhaps the most prominent biological science discipline to connect to data science. We investigate how genomics fits in with many of the other areas of data science, in terms of its data volume, velocity, and variety. Furthermore, we discuss how genomics may be able to leverage modeling (both physical and biological) to enhance predictive power, similar in a sense to what has been achieved in weather forecasting. Finally, we discuss how many data science ideas have been both imported to and exported from genomics. In particular, we explore how the HGP might have inspired many cultural practices that led to large-scale adoption of open-data standards.

We conclude by exploring some of the more urgent issues related to data, and how they are impacting data in genomics and other disciplines. Several of these issues do not relate to data analytics per se but are associated with the flow of data. In particular, we discuss how individual privacy concerns, more specifically data ownership, are central issues in many data-rich fields, and especially in genomics. We think grappling with several of these issues of data ownership and privacy will be central to scaling genomics to an even greater size in the future.

## Abbreviations

CASP: Critical Assessment of Protein Structure Prediction
CNN: Convolutional Neural Network
ENA: European Nucleotide Archive
HGP: Human Genome Project
HMM: Hidden Markov model
LDA: Latent Dirichlet allocation
